# Adipose‐Derived Extracellular Vesicles and Intercellular Crosstalk With Skeletal Muscle: Implications for Sarcopenic Obesity and Metabolic Dysregulation

**DOI:** 10.1111/obr.70005

**Published:** 2025-08-13

**Authors:** Michael Macleod, Joshua Price, Elpida Tsonou, David J. Baker, Kostas Tsintzas, Simon W. Jones

**Affiliations:** ^1^ Department of Inflammation and Ageing MRC‐Versus Arthritis Centre for Musculoskeletal Ageing Research, College of Medicine and Health, University of Birmingham Birmingham UK; ^2^ National Institute for Health and Care Research (NIHR) Birmingham Biomedical Research Centre Birmingham UK; ^3^ Bioscience Metabolism, Cardiovascular, Renal, and Metabolism (CVRM), AstraZeneca Cambridge UK; ^4^ MRC‐Versus Arthritis Centre for Musculoskeletal Ageing Research, School of Life Sciences University of Nottingham, Queen's Medical Centre Nottingham UK

**Keywords:** adipose tissue, crosstalk, extracellular vesicles, obesity, sarcopenia, skeletal muscle

## Abstract

Sarcopenic obesity, characterized by the concurrent presence of excess adiposity and diminished skeletal muscle mass and function, is closely linked to frailty, chronic inflammation, and insulin resistance. The increasing prevalence of sarcopenic obesity is driven by the global aging population, widespread adoption of sedentary lifestyles, and the ongoing obesity epidemic. Existing research describes a role for dysregulated crosstalk between adipose tissue and skeletal muscle tissue in driving sarcopenic obesity pathology, with recent evidence implying that extracellular vesicles (EVs, nano‐ to micro‐scale, lipid bilayer membrane‐delimited particles) have a significant role in facilitating intercellular communication to mediate critical tissue crosstalk. Given the significance of dysregulated tissue crosstalk in sarcopenic obesity pathology and the dysregulation of metabolism, the potential involvement of EVs has garnered considerable attention because of their scope as pharmacological targets and drug delivery vehicles, potentially leading to innovative therapeutic approaches. This review begins with an exploration of EV biology and the challenges associated with the standardization and execution of EV research. It then examines the impact of sarcopenic obesity risk factors on circulating and adipose‐derived EV profiles. Current understanding of the role of specific EV cargo, including microRNAs, proteins, and lipids, in mediating crosstalk between adipose tissue and skeletal muscle is critically evaluated. Finally, the potential of EV‐based therapeutics for treating sarcopenic obesity is discussed, along with recommendations for future research directions.

## 1 Sarcopenic Obesity and the Role of Adipose and Skeletal Muscle Crosstalk

The global prevalence of obesity, coupled with the rapidly aging population, is driving increased incidence of frailty and metabolic disease [1, 2]. It is estimated that, worldwide, between 4.5% and 11% of adults aged 60 and over present with concomitant obesity and relative reductions in skeletal muscle mass, quality, and function [3, 4], termed sarcopenic obesity [5]. Sarcopenic obesity is associated with chronic inflammation and impaired physical function and is often accompanied by insulin resistance [6]. Expert consensus states that, because of “pathogenic interaction” between excess adiposity and decreased muscle mass, sarcopenic obesity poses a “synergistically higher risk” for impaired physical and metabolic function than the cumulative risk of obesity and sarcopenia individually [5]. Sarcopenic obesity therefore presents significant personal and socioeconomic challenges [7].

Through investigating molecular mechanisms underlying sarcopenic obesity pathology, cellular crosstalk between adipose tissue (AT) and skeletal muscle (SkM) has emerged as a crucial research area. Although AT was classically considered to function solely as an energy storage organ, it is now well documented to have endocrine properties, secreting signaling molecules to induce effects systemically in organ‐to‐organ crosstalk [8]. Crucially, because SkM is an important site for glucose disposal, crosstalk between AT and SkM impacts not only physical function but also metabolic function [9]. The chronic inflammatory state induced by aging and obesity (inflammageing [1, 10]) promotes dysregulation of AT crosstalk with several tissues [11], including SkM [12], and thus plays a role in driving sarcopenic obesity and insulin resistance in a vicious cycle [13].

Previous studies evidence that crosstalk between AT and SkM is mediated by tissue‐specific cytokines, with adipokines being released into circulation and acting systemically [12, 14]. However, recent interest in the role of extracellular vesicles (EVs, nano‐ to micro‐scale, lipid bilayer membrane‐delimited particles that cannot replicate), which are generated by the majority of cell types [15], has presented an alternative paradigm in our understanding of tissue crosstalk complexity [16]. It has been proposed that EVs participate in crosstalk by transporting biological cargo (ribonucleic acid (RNA) species, deoxyribonucleic acid (DNA) species, proteins, and lipids) to induce functional effects in recipient cells upon delivery [17].

The focus of this review is EV‐mediated crosstalk between AT and SkM in humans and animal models. Herein, existing evidence on the role of EVs in AT–SkM crosstalk is critically discussed, aiming to unravel this aspect of the intricate pathophysiology of sarcopenic obesity and metabolic dysregulation.

## 2 EV Biology

The realization that EVs are implicated in a variety of biological processes and are released by most cell types has led to rapid growth of the EV field [15]. This has been accompanied by advancement in technologies facilitating EV research, yet standardization and utilization of these technologies are inconsistent. To rectify this, the International Society for Extracellular Vesicles (ISEV) has published guidance for EV researchers [15]. Despite this guidance, inconsistent terminology and variation in EV workflows still appear and make interpreting findings problematic. This section, using ISEV guidance, seeks to provide clarity around EV research methods to contextualize later discussion of EV‐mediated crosstalk.

**FIGURE 1:**
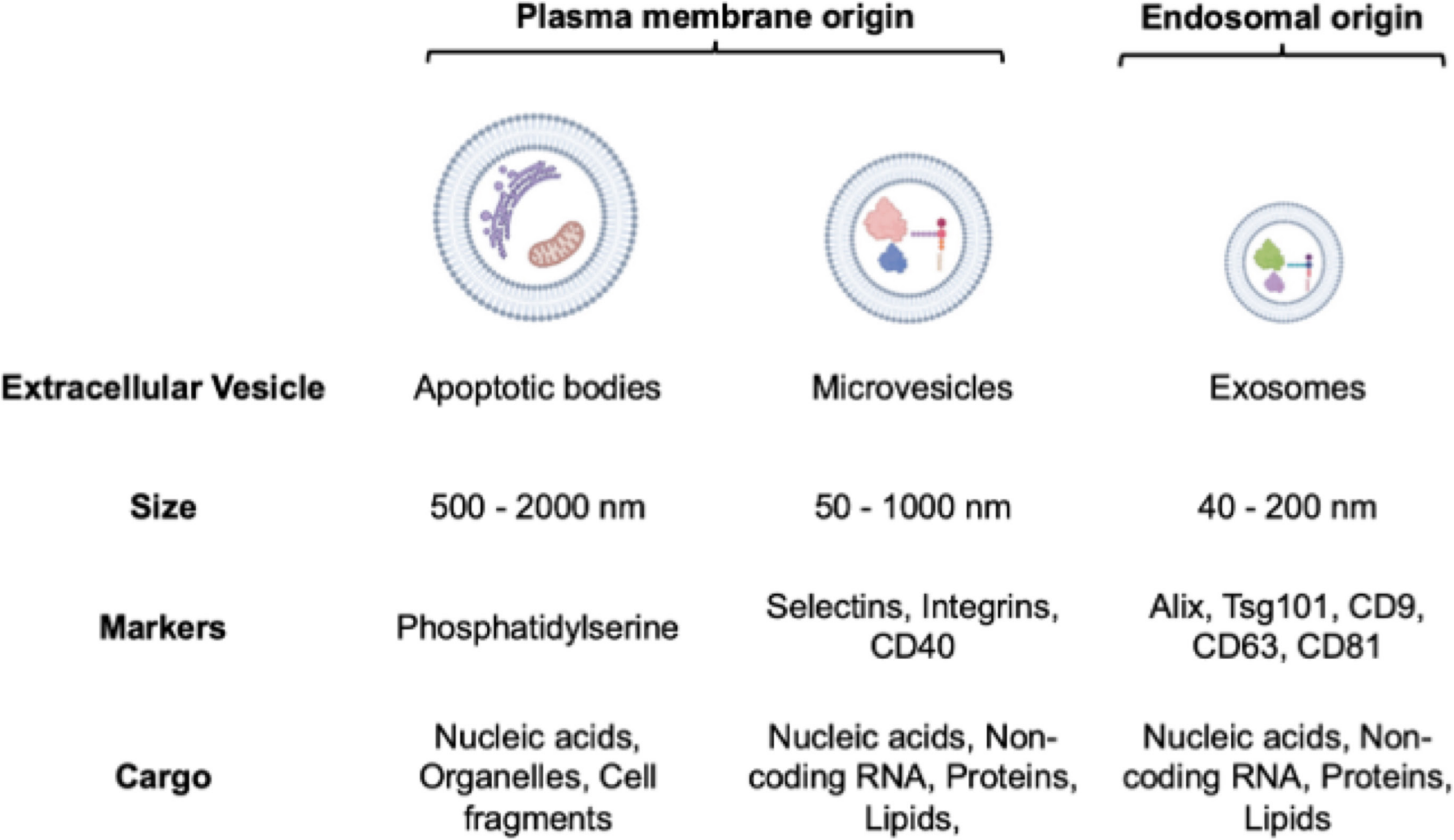
Classification of EVs based upon their origin. EVs are broadly categorized by their cellular origins as either from the plasma membrane (apoptotic bodies and microvesicles) or from the endosome (exosomes). Their size distributions, commonly identified markers, and cargo are shown. Created in BioRender. Macleod, M. (2025) https://BioRender.com/dahj2vz

EVs can be broadly split into two categories, endosomal origin (i.e., exosomes) or plasma membrane origin (i.e., microvesicles and apoptotic bodies) [15], as shown in Figure 1. Regarding nomenclature, the terms exosomes, microvesicles, microparticles, ectosomes, virus‐like particles, and pequeñas particulas have been widely used [18]. These were often arbitrarily chosen by authors or based on particle size [19]; however, it is now appreciated that EVs are highly heterogeneous and overlapping in size, cargo, and biological functions [20, 21]. These features alone, therefore, cannot determine whether EVs are endosomal‐ or plasma membrane‐derived [15]. Although putative markers of EV subtype exist (see Figure 1), these lack reliability across cell types [22]. Literature often continues to refer to EVs of unknown origin as exosomes, with evidence implying that “exosome” is perceived as a more desirable term by cell biologists, especially regarding EV‐based therapeutics [19]. However, for the purpose of this review, we will use the term EV, unless cellular origin is demonstrated.

Sources of EVs are numerous and varied, from biological fluids (urine, saliva, and plasma) to tissue explants and cell culture conditioned media. Generating concentrated EV samples from these sources can be complex, and because the likelihood of achieving a pure EV preparation is low (because of co‐isolates), the term “isolation” is discouraged, and instead EV samples are described as “separated.” Extensive reviews of EV separation methods have been published previously, highlighting the advantages, pitfalls, and suitability of each method [23, 24]. Following separation, characterization of sample to confirm EV enrichment and inform downstream experimental application is essential and has also been described previously [25]. Functionally, EV uptake by recipient cells is likely key; previous reviews elegantly describe mechanisms by which this occurs [26, 27]. Briefly, EVs can be separated from their source by differential ultracentrifugation (dUC), whereby stepwise centrifugations of increasing force deplete the sample of non–EV elements (i.e., cell debris and proteins); size exclusion chromatography (SEC), whereby the sample is passed through a porous column and eluted in fractions, each containing progressively smaller particles; or immunoaffinity‐based precipitation, whereby EV surface markers are used to bind and extract EVs from the sample. Once separated, EV‐enriched preparations can be characterized by numerous methods. Nanoparticle tracking analysis (NTA) uses microscopy to visualize and record EV size and concentration. Other methods use fluorescence‐based visualization via common EV surface markers or (nano)flow‐cytometry‐based single‐particle analysis to inform size and concentration. Alternatively, protein concentration can be useful to inform experimental dosage, but this lacks EV specificity. EVs can be imaged directly via transmission or scanning electron microscopy (TEM or SEM) to confirm presence within a sample, but additional methods are still required to determine size and concentration.

## 3 Relationship Between Sarcopenic Obesity–Related Lifestyle Conditions and EV Profile

Lifestyle factors (diet and physical activity levels) and related conditions (obesity and diabetes) have acute and chronic impacts on inflammation [28, 29, 30, 31]. Given that one mechanism by which inflammation acts is cytokine secretion [32], EV secretion and altered EV cargo likely also mediate inflammation. Indeed, numerous reviews support this, positioning EVs as vital effectors in inflammation and associated diseases [16, 33]. This section, summarized in Tables 1, 2, 3, 4, presents evidence that EV profiles are altered in the presence of conditions associated with sarcopenic obesity, potentially implying pathophysiological alterations in EV functionality. It should be noted that the relationships discussed below may be correlative in nature, and determining directionality is an ongoing challenge in EV research. As such, care is taken to not attribute causation when the data do not imply this. Additionally, as discussed, EV research methodology is variable throughout the literature. Where relevant, methodological differences have been highlighted and reviewed for comparative purposes; however, the main objective herein is to collate and critique findings. As the field develops and more research is published, comparative analysis of methodologies will become more feasible, but for the purposes of this review, Section 2 is provided to enable readers to make informed inferences about EV methodology in the works cited herein, thus enabling a greater breadth of research to be included.

### 3.1 Obesity

Given the current levels of physical inactivity and generally abundant food availability, obesity levels are pandemic. Obesity induces physiological stress characterized by pathological phenotypes; one such phenotype appears to be EV associated (Table 1).

**TABLE 1:** Summary of the impact of obesity on EV profile.

EV profile	EV source	EV isolation method	EV characterization	Effect on EV profile	Reference
Size and concentration	Human plasma	dUC	NTA, tetraspanin expression by flow cytometry	Small increase in EV count and no change in size	Amosse et al. [34]
	Human subcutaneous and visceral AT explants	dUC	NTA, immunoblotting for CD9, CD63, and syntenin‐1	Increased EV concentration	Camino et al. [35]
	Human adipose tissue explants	(d)UC	NTA, transmission electron microscopy, immunoblotting for ALIX, TSG101, and FLOT1	Increased EV concentration with increased BMI	Lazar *et al*. [36]
	Human subcutaneous and visceral AT explants	dUC	Fluorescent flow cytometry	Decrease in subcutaneous AT EV with waist circumference, and no change in visceral EV	Kranendonk et al. [37]
Protein	Human plasma and AT explants	dUC	NTA, immunoblotting for CD9, CD63, and syntenin‐1	Trends for elevated mimecan and TGFβI, and decreased syntenin‐1	Camino et al. [35]
	Murine AT (adipocyte fraction)	SEC and dUC	NTA, cryoTEM, and CD63 blotting (no clear presence of CD63 shown)	Alterations in 417 proteins	Kulaj et al. [38]
miRNA	Murine visceral AT	Centrifugation then ExoQuick	TEM and tetraspanin expression by immunoblotting	274 differentially expressed miRNA with obesity and specifically decrease in miR‐141‐3p with obesity	Dang et al. [39]
	3 T3‐L1 adipocytes	dUC	Identify CD63 and FABP4 (not shown in the manuscript or Supporting Information)	Elevated miR‐802‐5p with obesity	Wen et al. [40]
	Murine adipocytes	dUC	TEM and immunoblotting of MFG‐E8	Elevated miR‐155 with obesity	Zhang et al. [41]
	Murine quadriceps muscle tissue	dUC	TEM with CD81 immunogold labeling and immunoblotting for CD63	Elevated miR‐16 with obesity	Jalabert et al. [42]
	Adolescent human subcutaneous and visceral adipose tissue	Centrifugation then ExoQuick	NTA and flow cytometry tetraspanin detection	55 differentially expressed miRNA with obesity	Ferrante et al. [43]
	Human plasma and serum	ExoQuick and immunomagnetic positive selection of FABP4 + EV	None	56 relevant differentially expressed miRNA following bariatric surgery weight loss	Hubal et al. [44]

Initial work by Amosse et al. claimed that circulating EV counts increase with BMI, as assessed by flow cytometry and NTA; however, differences were minimal, and inter‐individual variation was high [34]. BMI definitions in this study were narrow (control BMI < 27, overweight BMI 27–30, and obese BMI > 30); thus, future work should sample participants across a wider BMI range to elicit more convincing data. Regarding adipose‐derived EVs specifically, Camino et al. compared EV yield from individuals of lean and obese BMI (average BMI 26 (lean) and 48 (obese)) and observed that the number of EVs released from obese AT was two orders of magnitude greater than from lean, and EVs from obese donors were larger than those from lean [35]. Lazar et al. used NTA to show a strong positive correlation between BMI and human AT EV number (*R* ^2^ = 0.76) [36]. Intriguingly, the opposite was found by Kranendonk et al., who showed an inverse correlation between waist circumference and the number of EVs released by subcutaneous AT (*R* ^2^ = −0.61) [37]. However, UC protocols and EV quantification methodology differ between these papers (overnight UC at 100,000 × *g* [36] vs. dUC with a final 100,000 × *g* centrifugation for 2 h [37], and NTA [36] versus fluorescent flow cytometry [37]), potentially explaining contradictory findings and emphasizing both the challenges of EV research and the importance of interrogation of research methodology prior to literature comparisons.

EV cargo also appears to be altered in obesity, likely more significant for functional conclusions. EVs are known to contain protein [45]; thus, Camino et al. conducted proteomic analysis, finding that visceral AT‐derived EVs from individuals with obesity trended toward decreased syntenin1 but elevated mimecan and transforming growth factor‐beta‐induced protein (TGFβI), compared to those from lean individuals [35]. The authors also separated EVs from plasma, finding similar results in circulation. However, these results lacked statistical significance because of large inter‐individual variation. More convincing, albeit nonhuman, data were obtained by Kulaj et al. using a high‐fat diet (HFD) mouse model of obesity and performing proteomic analysis on AT‐derived EVs [38]. The work showed clear differences in EV protein cargo from lean and obese mice, with 417 proteins altered. In the obese condition, proteins implicated in inflammation and insulin resistance (LEP, RBP4, and MHC Class‐II members) were elevated, whereas EVs from lean animals contained elevated levels of proteins associated with insulin sensitivity and anti‐inflammatory pathways (ADIPOQ, GPC5, INSR, MRC1, and CD163). This work is strengthened by the use of both dUC and SEC to separate EVs, with no apparent difference between preparations.

MicroRNAs (miRNAs; small, non‐coding RNA molecules that regulate post‐transcriptional gene expression) are also a key component of EV cargo [46]. Multiple studies have reported alterations in EV miRNA cargo in obesity. Dang et al. compared the miRNA content of AT‐derived EVs from lean and obese mice, finding 274 differentially expressed miRNAs in obesity [39]. Of interest was miR‐141‐3p, which was downregulated in EVs from obese mice, driving reductions in hepatocyte glucose uptake in vitro. Wen et al. used a murine adipocyte cell line (3 T3‐L1 cells differentiated into adipocytes) to illustrate that hypertrophic (obese) adipocytes (generated by palmitate treatment) release EVs enriched in miR‐802‐5p, which in turn induce insulin resistance in murine cardiomyocytes [40]. Robust increases in miR‐155 within adipocyte‐derived EVs from HFD mice were demonstrated by Zhang et al. [41] This elevation drove macrophages toward the “pro‐inflammatory” M1 phenotype. Jalabert et al. demonstrated that HFD‐induced obesity alters the miRNA cargo of SkM‐derived EVs [42]. EVs from quadriceps tissue of HFD mice were enriched for miR‐16 compared to EVs from control animals, with implications in PI3K/AKT signaling. Human evidence for altered EV miRNA cargo also exists. Ferrante et al. profiled the miRNA cargo of EVs derived from lean and obese visceral AT, finding 55 differentially expressed mature miRNAs between conditions [43]. Highly targeted pathways from these differentially expressed miRNAs included TGF‐β and Wnt/B‐catenin pathways, implicated in chronic inflammation [47]. Participants in this study were recruited from an adolescent bariatric or abdominal surgical procedure; given that obesity‐associated chronic inflammation increases with aging (inflammageing), the observed differential expression of miRNAs in young individuals may be exacerbated in older adults. Elegant work by Hubal et al. implies that changes in adipocyte‐derived EV miRNA cargo in circulation are directly related to BMI as opposed to individual variability [44]. The authors performed a longitudinal analysis of patients undergoing gastric bypass surgery and revealed altered EV miRNA cargo (related to insulin signaling pathways) post‐surgery compared to pre‐surgery. Participants experienced decreases in BMI and insulin resistance post‐surgery, implying a functional effect of EV miRNA changes; however, this study fails to report any EV characterization.

Together, these studies largely evidence that, either in circulation or derived from tissue explants, obesity is associated with increased EV concentration, modulated protein cargo, and differential expression of specific miRNA cargo.

### 3.2 Type 2 Diabetes

Type 2 diabetes mellitus (T2DM) is an increasingly common metabolic condition characterized by an inability to regulate plasma glucose levels because of tissue insensitivity to insulin and beta cell dysfunction. T2DM induces chronic, low‐level physiological stress reflected by changes in tissue secretome [48, 49]; hence, the EV profile is likely also reflective of this disease state (Table 2). Indeed, NTA of circulating EVs from a mouse model of T2DM (Lepr^db/db^) showed significantly increased EV size and concentration compared to control animals [50]. The same study performed similar work in diabetic and nondiabetic human participants, again finding increased size and concentration of circulating EVs with diabetes. However, work by Cheng et al. shows no difference in EV size or concentration between diabetic (again, Lepr^db/db^) and nondiabetic mice via NTA [51]. This difference may be explained by and highlights the challenges of EV separation methodology. The former study uses SEC to isolate EVs [50], whereas the latter uses density‐gradient UC [51]; given the previously referenced [23, 24, 25] limitations of each method and the limitations of NTA as a measurement tool, comparison of results should be performed with caution. Human work concurs, however, that EV number can be altered in prediabetes and T2DM. Both Kobayashi et al. and Freeman et al. found that individuals with either prediabetes (determined by impaired glucose tolerance and elevated plasma triglyceride levels) or T2DM had higher circulating EV concentration versus individuals with normal glucose tolerance and triglyceride levels [52, 53]. The work by Freeman et al. also includes a second cohort of BMI‐matched participants with and without T2DM; in this cohort, the authors stratified their analysis by race, finding that T2DM was only associated with increased circulating EV concentration in White participants but that the effect was not present in African American participants when matched for BMI. This highlights the critical issue of concomitant obesity and diabetes (“diabesity”) in confounding findings in this field. Notably, Nunez Lopez et al. use BMI‐matched cohorts of individuals with and without T2DM and find no change in concentration of circulating EVs [54].

**TABLE 2:** Summary of the impact of T2DM on EV profile.

EV profile	EV source	EV isolation method	EV characterization	Effect on EV profile	Reference
Size and concentration	Murine and human serum	SEC or ExoQuick	NTA, cryoEM, immunoblotting for CD63 and ALIX, and colorimetric confirmation of depleted lipoprotein contamination	Increased size and concentration of EV with diabetes, in both mice and humans	Gustafson et al. [50]
	Murine serum	Density‐gradient UC	NTA and immunoblotting for FLOT1 and TSG101	No change in EV concentration with diabetes	Cheng et al. [51]
	Human plasma	SEC	Flow cytometry, immunoblotting for annexin V, and CD9	Increased EV concentration with (pre)diabetes	Kobayashi et al. [52]
	Human plasma	ExoQuick or dUC	NTA, TEM, immunoblotting for ALIX, FLOT1, and TSG101	Increased EV concentration with diabetes	Freeman et al. [53]
Protein	Human plasma	ExoQuick or dUC	NTA, TEM, immunoblotting for ALIX, FLOT1, and TSG101	Altered EV protein cargo with diabetes	Freeman et al. [53]
	Murine and human serum	SEC or ExoQuick	NTA, cryoEM, immunoblotting for CD63 and ALIX, and colorimetric confirmation of depleted lipoprotein contamination	Increased protein cargo implicated in activating MAPK and Rho kinase	Gustafson et al. [50]
	Human serum	EVtrap	NTA and SEM	No change in EV concentration, 308 proteins and 191 phosphoproteins differentially expressed with diabetes, and impaired phosphorylation of EV proteins with diabetes	Nunez Lopez et al. [54]
	Human subcutaneous and visceral AT explants	dUC	NTA, immunoblotting for CD9, CD63, syntenin‐1	TGFβI reduced in EV from obese individuals with diabetes	Camino et al. [35]
miRNA	miRNA source: human serum	n/a	n/a	Elevated expression of seven diabetes‐associated miRNAs with diabetes	Kong et al. [55]
	miRNA source: human serum	ExoQuick	n/a	Unique clustering of miRNAs in control, diabetes, and metabolic syndrome	Karolina et al. [56]
	“circulating”	Not stated	Not stated	> 20 miRNAs differentially expressed between prediabetic and diabetic patients	Park et al. [57]
	Human serum	Centrifugation then ExoQuick	NTA	miR‐10b and miR‐223‐3p are biomarkers of diabetes	Parrizas et al. [58]
	Human plasma	centrifugation and miRCURY Exosome Isolation Kit—serum and plasma	TEM, NTA, immunoblotting for CD9, CD81, and HSP70	Eight miRNAs differentially expressed in diabetes	Masi et al. [59]
	Human serum	miRCURY Exosome Isolation Kit—serum and plasma	NTA, immunoblotting for CD9, ALIX, and APOA1 in the EV‐pellet and the supernatant	Six miRNAs differentially expressed in the EV fraction with diabetes but no difference in total circulating miRNA	Katayama et al. [60]

EV cargo also appears to be different in T2DM, potentially altering the physiological function of EVs. Freeman et al. found an increase in circulating EVs in diabetic versus normoglycemic individuals but critically also found alterations in EV protein cargo implicated in insulin signaling pathways in diabetic individuals [53]. Work by Gustafson et al. shows that, in both a T2DM mouse model and in human patients, EV protein cargo is altered significantly in the disease state, modulating the mitogen‐activated protein kinase (MAPK) and Rho kinase pathways [50]. Proteomic analysis of circulating EVs from BMI‐matched individuals with normal glucose tolerance, prediabetes, and T2DM by Nunez Lopez et al. showed distinct EV protein cargo across the three conditions, although no change in concentration was found [54]. Notably, EVs from diabetic individuals exhibited 308 differentially expressed proteins and 191 differentially expressed phosphoproteins versus the control and prediabetes conditions. The authors analyzed protein: phosphoprotein ratios, with EVs from diabetic individuals showing impaired phosphorylation of protein cargo implicated in immune, inflammation, and metabolic pathways. The use of BMI‐matched groups in this study is critical in isolating the effect of T2DM, given the close link between T2DM and obesity. On this, Camino et al. showed that TGFβI was depleted in EVs from T2DM patients with obesity when compared to both lean *and* obese normoglycemic individuals [35], illustrating that, despite pathophysiological overlap, diabetes and obesity are reflected in distinct EV cargo modulations. To our knowledge, no other work has separated the effects of T2DM and obesity in this manner; thus, given the confounding effects of diabesity, future work should seek to stratify their analyses across both conditions, if participant sample allows.

The miRNA cargo of EVs also appears to be modulated by T2DM. A thorough review by Chao et al. summarized the literature regarding circulating miRNA levels in diabetes, concluding that, despite numerous inconsistencies, two microRNAs, miR‐126 and miR‐34a, show robust diabetes‐associated elevations [61]. However, much of their cited work does not demonstrate that the miRNAs are EV associated. Circulating miRNAs are likely packaged into EVs for protection, as blood is rich in RNases and thus free miRNAs are likely degraded [62]. Nevertheless, without separating EVs prior to miRNA analysis, conclusions about EV cargo are confounded. For example, Kong et al. and Karolina et al. profiled circulating miRNAs in diabetes and metabolic syndrome patients, finding distinct miRNA expression patterns compared to healthy controls, but neither group demonstrated EV association in their analysis [55, 56]. Some original work, however, does make an attempt to directly profile EV‐specific miRNAs in diabetes. Park et al. used next‐generation sequencing to reveal differential expression of over 20 miRNAs within circulating EVs of individuals with T2DM compared to those with prediabetes [57]. Parrizas et al. examined miRNAs in circulating EVs (separated by differential centrifugation and ExoQuick kit) from prediabetic patients, before following up in those that developed T2DM within 4 years, showing that miR‐10b and miR‐223‐3p, associated with insulin signaling, function as biomarkers of transition from prediabetes to T2DM [58]. Minimal EV characterization was performed, or at least published, in these studies [57, 58]; thus, the question of whether these miRNAs are EV associated persists. However, Masi et al. showed eight EV‐associated miRNAs to be altered in circulating EVs from T2DM patients compared to BMI‐matched normoglycemic controls [59]. EV separation in this study is performed by the miRCURY exosome isolation kit (exiqon), so there is potential confounding of results by enriching for specific EV populations that this kit selects for; however, thorough characterization of the resulting EV population via TEM, NTA, and Western blotting for classical EV markers (CD9, CD81, and HSP70) demonstrates that the miRNAs identified in this work are likely EV associated. Katayama et al. also used the miRCURY exosome isolation kit (exiqon) to examine circulating EV‐ and non–EV‐associated miRNA profiles in BMI‐matched diabetic and nondiabetic patients; no difference existed in total circulating miRNA profile, but six differentially expressed miRNAs were present in EVs, implying specific modulation of the EV‐associated miRNAs [60]. EV characterization in this study was also strong, including immunoblotting comparisons of serum and “EV‐enriched” fractions, with the latter displaying elevated expression of ALIX and CD9 (EV markers) and reduced expression of APOA1 (a lipoprotein marker; lipoprotein co‐isolation with EV is a common confounder) compared to corresponding serum samples, evidencing that these miRNAs are EV associated.

In summary, numerous studies demonstrate increased circulating EV concentration with T2DM, significant differential expression of EV protein cargo, and altered circulating miRNA profiles in T2DM. It must be noted, however, that diabesity confounds some of the discussed work and extensive additional research is required to separate the effects of obesity and T2DM on EV profiles. Additionally, because of the challenges presented by EV research, few studies present strong evidence that miRNAs in question are EV associated. Despite this, Masi et al. and Katayama et al. do demonstrate that EV‐associated miRNA cargo is modulated in the T2DM disease state, demonstrating the potential of EVs as biomarkers for T2DM (if the effect can be distinguished from and obesity‐driven mechanism) and inviting further work to establish whether disease progression is EV mediated.

### 3.3 Age

Aging can drive tissue dysfunction through several biological processes termed hallmarks of aging (cellular senescence, epigenetic changes, mitochondrial dysfunction, and increased pro‐inflammatory cytokine release). A growing body of evidence implicates EV‐mediated mechanisms in dysfunctional aging (Table 3). Rodent studies have elucidated mixed findings linking age with EV number; one study showed no difference between young and old animals [63], but other studies have shown either increases in circulating EVs with age [64] or mixed results depending on sample separation and analysis methods [65]. Similar inconsistency is present in human evidence; Eitan et al. found longitudinal and cross‐sectional age‐related decline in circulating EV concentration, attributed to increased uptake of EVs by immune cells such that fewer remain in circulation, but found no change in EV size [66]. In contrast, numerous groups find no correlation between age and EV count, measured by NTA [67, 68, 69] or flow cytometry [70].

**TABLE 3:** Summary of the impact of age on EV profile.

EV profile	EV source	EV isolation method	EV characterization	Effect on EV profile	Reference
Size and concentration	Murine serum	Centrifugation (10,000 × *g* for 10 min) with added total exosome isolation reagent	NTA and immunoblotting for CD63 and HSP70	No change in EV size or concentration with age	Tsukamoto et al. [63]
	Murine serum	SEC	NTA and flow cytometry for CD63 and CD81	Increased EV concentration with age	Sahu et al. [64]
	Murine plasma	SEC or ExoQuick	NTA, TEM, flow cytometry for CD63, immunoblotting for TSG101, CD63, CD81, calnexin, and lipoprotein markers	Reduced EV size and concentration with age by NTA but increased CD63 + EV and increased immunoblot levels of CD63, CD81 and TSG101, and reduced lipoprotein contamination with age	Alibhai et al. [65]
	Human plasma	ExoQuick	NTA, TEM, immunoblotting for CD9, FLOT1, and TSG101	No change in EV size with age, but EV concentration declined with age (proposed to be due to an increase in EV uptake by immune cells with age, leaving fewer remaining in the circulation)	Eitan et al. [66]
	Human plasma	Centrifugation (13,000 × *g* and 20,000 × *g*)	NTA	No correlation between age and EV size	Alberro et al. [67]
	Human plasma	Centrifugation then SEC and UC	NTA, TEM, immunoblotting for ALIX, TSG101, syntenin‐1, CD9, CD63, CD81, albumin, and APOA1	No change in EV size or concentration with age	Xhuti et al. [68]
	Human plasma	Density cushion separation of lipoproteins followed by SEC	NTA, presence of CD9, CD63, and CD81	No change in EV size or concentration with age	Grenier‐Pleau et al. [69]
	Human plasma	ExoQuick	Dynamic light scatter, flow cytometry for CD9, CD63, CD81, CD41a, and other tetraspanins	No change in EV size or concentration with age	Zhang et al. [70]
Protein	Human plasma	ExoQuick	NTA, TEM, immunoblotting for CD9, FLOT1, and TSG101	Decrease in apoptosis proteins but increase in immune‐related proteins, with age	Eitan et al. [66]
	Human plasma	dUC	NTA, EM, CD63 immunoprecipitation	Decrease in galectin‐3 with age	Weilner et al. [71]
	Human plasma	Density cushion separation of lipoproteins followed by SEC	NTA, presence of CD9, CD63, and CD81	No change in total protein concentration but changes in specific protein cargo with age	Grenier‐Pleau et al. [69]
miRNA	Murine serum	ExoQuick	Photon correlation spectroscopy and atomic force microscopy	34 differentially expressed miRNAs with age	Wang et al. [72]
	Murine plasma	SEC or ExoQuick	NTA, TEM, flow cytometry for CD63, immunoblotting for TSG101, CD63, CD81, calnexin, and lipoprotein markers	13 differentially expressed miRNAs with age	Alibhai et al. [65]
	Murine serum	Centrifugation (10,000 × *g* for 10 min) with added total exosome isolation reagent	NTA and immunoblotting for CD63 and HSP70	15 upregulated miRNAs with age, specifically miR‐192	Tsukamoto et al. [63]
	Murine ex vivo *peri‐*gonadal white adipose tissue	ExoQuick	NTA and immunoblotting for FLOT1 and FABP4	131 differentially expressed miRNAs between young and middle aged mice	Cho et al. [73]
	Murine plasma	UC	NTA and cryoTEM	82% of plasma‐EV miRNAs detected in young animals were demonstrated increased expression in old animals	Kern et al. [74]
	Human plasma and muscle cell line	centrifugation then SEC and UC	NTA, TEM, immunoblotting for ALIX, TSG101, syntenin‐1, CD9, CD63, CD81, albumin, APOA1	differentially expressed miRNAs from plasma and muscle EVs with age	Xhuti et al. [68]
	Human and murine primary adipose progenitor cells	UC	TEM, immunoblotting for TSG101, CD63, and calnexin	Decline in miR‐145 with age	Zhou et al. [75]
	Human saliva	Centrifugation (10,000 × *g* for 1 h) with total exosome isolation reagent	None	Increased miR‐24‐3p with age	Machida et al. [76]

Research into altered EV protein cargo with age is sparse, but Eitan et al. followed up size and concentration characterization with ELISA assays on EV lysates, finding age‐associated declines in proteins implicated in apoptotic processes but increases in immune‐related proteins both cross‐sectionally and longitudinally (5‐year follow‐up) [66]. Another group identified a decline in galectin‐3 in plasma EVs of older adults compared to young individuals, which correlated with impaired osteogenic inductivity [71]. Grenier‐Pleau et al. also supported their size and concentration data with proteomic analyses, revealing no change in total protein content with age, but significant changes in specific protein cargo and circulating EV source (increased pancreatic EVs and decreased hepatic EVs with age) are reported [69].

Comparatively more research has investigated age‐associated changes in EV miRNA cargo; however, much of this work utilizes murine models. Wang et al. profiled miRNA cargo of circulating EVs from young and aged mice, revealing 34 differentially expressed miRNAs with age (19 upregulated and 15 downregulated) [72]. Another study also profiled young and old circulating mouse EVs, finding nine miRNAs expressed only in EVs from old animals and four only in those from young animals [65]. Of interest, miRNAs upregulated with age‐targeted transcriptional regulation and nucleotide binding, specifically mediated via MAPK. Tsukamoto et al. presented RNA‐sequencing data showing 15 upregulated miRNAs in plasma EVs from aged mice compared to those from young mice [63]. Targeted follow‐up revealed miR‐192 to be crucial in driving age‐related immune adaptations and cytokine signaling processes. Indeed, administering anti‐IL6 antibody to aged animals or knocking out IL‐6 reduced EV miR‐192, whereas recombinant IL‐6 administration increased miR‐192 in plasma EVs of aged mice. The authors suggest that miR‐192 reduces pro‐inflammatory cytokines, attenuating inflammageing. Recent work profiled 599 miRNAs in young and middle‐aged mice, finding 131 differentially expressed with age [73]. Even on a normal diet (as opposed to HFD), aging mice demonstrated altered expression of miRNAs associated not only with aging (miR‐34a and miR‐15a) but also with obesity (miR‐150 and miR‐2141), implying a shared, concurrent mechanism across diet and aging, relevant for sarcopenic obesity. Kern et al. demonstrated that 82% of plasma EV‐miRNAs increase in expression with age, notably to a significantly greater extent than the non–EV fraction of circulating miRNA [74]. The authors specifically identify the miR‐29 family in regulating aging processes in adipose tissue. Xhuti et al. assessed miRNA content of plasma EVs from young and old individuals and used an in vitro model of aging (late‐stage passage to increase cellular senescence) in a human myoblast cell line differentiated into myotubes and revealed a significant decline in a number of canonical muscle‐related miRNAs, miR‐1, miR‐206, and miR‐133a, as well as various other miRNAs, in plasma‐ and myotube‐derived EVs [68]. Recent work by Zhou et al. demonstrates a robust age‐associated decline in miR‐145 cargo in adipose progenitor cell EVs in both mice and humans, mechanistically linking this to increased age‐associated inflammation [75]. Aside from this work, there is a scarcity of in vivo human data validating EV miRNA cargo changes with age; however, Machida et al. do show a small yet significant increase in salivary EV miR‐24‐3p with age [76]. All other candidate miRNA aging biomarkers identified by the authors showed no change with age.

When taken together, these publications demonstrate a lack of correlation between age and EV concentration, but changes in EV protein and miRNA cargo do appear to be present in aging, although primary in vivo human validation of this is sparse.

### 3.4 Physical Activity

Physical activity, both acutely and as chronic exercise training, has myriad beneficial effects, especially in the contexts of sarcopenic obesity and metabolic dysregulation [77]. Given the emergence of EVs as cell‐to‐cell communicators, recent work implies a role for EVs in mediating exercise benefits (Table 4). However, inherent issues with EV workflows, coupled with exercise‐related variables (timescale, intensity, modality, fitness levels, assessment methods, and sampling time), mean that the literature is largely inconclusive on the effects of exercise on EVs. For example, Oliveira et al. observed a decrease in circulating EV size in rats immediately after vigorous exercise compared to moderate exercise, but an increase in circulating EV concentration as a result of exercise, irrespective of intensity [78]. This study does not report animal age, but reported weight shows the animals, if adult, to be normal weight (not obese). Human work contains similar contradictions, with one study (Whitham et al.) finding an increase in concentration, but not size, of EVs immediately after exercise, which returned to baseline 4 h post‐exercise [79], whereas another study (Rigamonti et al.) showed that total circulating EVs decline immediately post‐exercise, and this is maintained at 3 and 24 h post‐exercise [80]. Exercise protocols differed between the studies (progressive cycling [79] vs. fixed‐intensity running [80]), potentially explaining differences in results. Additionally, Whitham et al. used only lean participants (average BMI 23.9) whereas Rigamonti et al. used both lean and obese participants. Further studies show no effect of exercise on EV size or concentration; Lovett et al. used downhill running and plyometrics but observed no change in size or concentration of plasma EVs from healthy young adults (potentially because of size‐based EV separation methods) [81], while Doncheva et al. used continuous, 45‐min cycling in middle‐aged adults with an average BMI of 26 and observed an increase in CD63+ particles within the EV preparation, with a trend for increased CD9 and CD81, implying increased EV within the sample following exercise [82]. Chong et al. also found increased EV concentration, but not size, following 20 min of cycling in both young and old healthy males [83].

**TABLE 4:** Summary of the impact of physical activity on EV profile.

EV profile	EV source	EV isolation method	EV characterization	Effect on EV profile	Reference
Size and concentration	Murine serum	ExoQuick	Tunable resistive pulse sensing, immunoelectron microscopy, immunoblotting for CD63, APOA‐IV, and calnexin	Increase in EV concentration after exercise and decrease in EV size after vigorous exercise	Oliveira et al. [78]
	Human plasma	Centrifugation	NTA and cryoEM	Increase in EV concentration after exercise, returned to baseline by 4 h	Whitham et al. [79]
	Human plasma	UC	NTA and flow cytometry for cell‐specific markers	EV concentration reduced immediately, 3 h and 24 h post‐exercise	Rigamonti et al. [80]
	Human plasma	SEC	NTA and TEM	No change in EV size or concentration following exercise	Lovett et al. [81]
	Human plasma	exoEasy Midi spin membrane affinity columns and SEC	NTA, immunoblotting for CD9, CD63, CD81, APOA1, and CD41	Increased CD63 in plasma EV preps following exercise and trend for increased CD9 and CD81	Doncheva et al. [82]
	Human plasma	dUC	Resistive pulse sensing, TSG101, syntenin‐1, and APOA1	Increased EV concentration following exercise	Chong et al. [83]
Protein	Electrical pulse stimulated (EPS) myotube conditioned media	Centrifugation with Centricon‐70 Plus 100 kDa centrifugal filter columns	NTA, TEM, immunoblotting for CD9, CD63, HSP70, calnexin, flow cytometry for CD9, CD63, and CD81	Differential expression of 170 EV proteins following EPS	Aas et al. [84]
	Human plasma	Centrifugation	NTA and cryoEM	322 EV proteins elevated immediately following exercise and only three remain elevated by 4 h post‐exercise	Whitham et al. [79]
	Human plasma	dUC	Resistive pulse sensing, TSG101, syntenin‐1, and APOA1	13 EV proteins differentially expressed following exercise	Chong et al. [83]
miRNA	Murine serum	ExoQuick	Tunable resistive pulse sensing, immunoelectron microscopy, immunoblotting for CD63, APOA‐IV, and calnexin	12 miRNAs differentially expressed following exercise	Oliveira et al. [78]
	Electrical pulse–stimulated (EPS) myotube conditioned media	Centrifugation with Centricon‐70 Plus 100‐kDa centrifugal filter columns	NTA, TEM, immunoblotting for CD9, CD63, HSP70, calnexin, flow cytometry for CD9, CD63, and CD81	57 miRNAs detectable in myotube EV following EPS versus without EPS	Aas et al. [84]
	Human plasma	SEC	NTA and TEM	Decline in miR‐31 24 h post‐exercise	Lovett et al. [81]
	Human plasma	exoEasy Midi spin membrane affinity columns, and SEC	NTA, immunoblotting for CD9, CD63, CD81, APOA1, and CD41	69% increase in EV miRNA after acute exercise, but no chronic change follows a 12‐week training period	Doncheva et al. [82]
	Human plasma	Centrifugation then SEC	NTA, TEM, immunoblotting for ALIX, TSG101, syntenin‐1, CD9, CD63, CD81, albumin, and APOA1	Several miRNAs decline with age, but resistance training in older adults causes miRNAs to trend toward the younger phenotype	Xhuti et al. [68]

EV protein cargo in physical activity is relatively understudied. In vitro work by Aas et al. showed that electrical pulse stimulation (EPS, an in vitro mimetic of muscular contraction) induced differential expression of 170 proteins in the cargo of EVs released by primary human myotubes isolated from middle‐aged females with obesity and T2DM [84]. Building on this, proteomic analysis of human EV cargo pre‐ and post‐exercise was conducted by Whitham et al., unveiling 322 differentially expressed proteins (majority upregulated) in circulating EVs immediately post‐exercise (60‐min progressive cycling) in healthy young adults [79]. The most highly targeted biological process by differentially expressed proteins was glycolysis. Intriguingly, only three proteins remained differentially expressed 4 h post‐exercise, implying transience in this effect. Chong et al. employed a shorter, 20‐min cycling protocol, finding 13 differentially expressed EV proteins after exercise [83]. Participants were split into “fit” and “unfit” groups; the “unfit” group demonstrated elevated levels of four proteins, whereas 10 proteins were elevated in the “fit” group. Of note, only S100A9 was uniquely elevated in the “unfit” group; the role of this protein in mediating inflammation has been elegantly reviewed previously [85], with emerging evidence now linking elevated S100A9 to skeletal muscle mitochondrial fragmentation [86], pancreatic cancer–associated cachexia [87], sarcopenia in kidney disease patients [88], and insulin‐independent anti‐diabetic effects in Type 1 diabetes [89]. Crucially, the exercise‐induced elevation of S100A9 in the “unfit” group represents an immediate post‐exercise response; the authors did not include later time points; thus, the increase may be transient. Nevertheless, future interrogation of EV‐associated S100A9 response to exercise may reveal novel insights into skeletal muscle homeostasis. Collectively, this work indicates differences in EV protein cargo due to physical activity and fitness level. The described differential effects of exercise on EV protein cargo in “fit” and “unfit” cohorts are relevant for individuals with sarcopenic obesity, given that impaired fitness and low activity levels are both risk factors for the development of, and characteristics of individuals with, sarcopenic obesity. Future work should aim to include individuals with a range of fitness levels in their work to further delineate the impact of exercise on EV profile.

Research profiling EV miRNA cargo in the context of physical activity is more extensive. In non‐obese rodents, acute aerobic exercise induced differential expression of 12 miRNAs implicated in MAPK regulation [78]. An in vitro study on primary human myotubes from middle‐aged females with obesity and T2DM revealed that EPS caused 57 miRNAs to be detectable in secreted EVs that were not present without EPS [84]. In vivo human trials also demonstrated an exercise effect; muscle‐damaging downhill running and plyometrics caused a decline in EV miR‐31, a regulator of satellite cell activator Myf5, in healthy young adults [81]. Doncheva et al. studied middle‐aged adults with an average BMI of 26 and found a 69% increase in EV‐associated miRNA expression following acute exercise, but there was no chronic alteration to EV miRNAs across 12 weeks of mixed‐modality training, supporting the concept that exercise‐induced changes in EV cargo are transient [79, 80, 81, 82]. In contrast, Xhuti et al. showed that following a 12‐week resistance training program, chronic alterations in circulating EV miRNAs can be achieved. Training partially restored a young phenotype in the circulating EV miRNA profile of older adults, demonstrating an EV‐associated marker of the geroprotective effects of resistance training [68]. These contrasting findings regarding chronic changes in EV profile with exercise may be explained by differences in participant age, adherence to prescribed exercise programs, or exercise modality. Nonetheless, future investigation into the effects of chronic exercise training on EV profile is both warranted to enhance understanding.

More work is required to elucidate the effect of exercise on EV profile and cargo due to significant variation in exercise protocols and EV methodology within the literature, but evidence implies that physical activity transiently, and potentially chronically following a training period, influences EV profile and cargo and thus may constitute a novel mechanism mediating the beneficial effects of exercise for individuals with sarcopenic obesity and metabolic disease.

### 3.5 Summary

As evidenced throughout this section, sarcopenic obesity–related conditions and lifestyle factors are associated with an altered EV profile (size, concentration, and cargo). Recent work also demonstrates that sarcopenia constitutes a unique EV proteome that holds diagnostic biomarker potential and warrants further mechanistic investigation [90]. It is thus reasonable to hypothesize that these altered EVs functionally participate in the dysregulated crosstalk that appears critical to sarcopenic obesity pathophysiology and may promote disease progression.

## 4 EV microRNA‐Mediated AT–SkM Crosstalk

As the most widely researched component of EV cargo, it is largely accepted that EV‐associated miRNAs participate mechanistically in cellular crosstalk. These short chains of nucleic acids (approximately 22 nucleotides) predominantly function by mediating gene expression post‐transcriptionally [91]. Conflicting evidence exists on whether miRNAs are mainly transported systemically by EVs or by association with the protein argonaute‐2 [46, 92]; however, as discussed in Section 3.2, strong evidence [59, 60] does now exist to demonstrate modulation of EV‐associated miRNA expression in disease (T2DM). Additionally, multiple studies demonstrate a necessity for EV carriers in facilitating functional miRNA‐mediated crosstalk, potentially because of protection from degradation [62], or enabling uptake and action at target cells [93]. For example, Thomou et al. showed that adipose‐derived, EV‐associated miR‐99b downregulates Fgf21, reducing insulin sensitivity in hepatocytes, but incubation of hepatocytes with “naked” miR‐99b had no effect [94]. The authors also elegantly demonstrate a crucial role for AT in contributing to total circulating EV‐miRNA levels; mice with an AT‐specific knockout of Dicer, the miRNA‐processing enzyme, exhibited a significantly depleted number of detectable EV‐associated miRNA by RT‐qPCR. There was also a reduction in total circulating serum miRNA but to a lesser extent than the observed reduction in EV‐associated miRNA, implying that AT preferentially contributes to the EV‐associated miRNA fraction. Other work shows that adipose EVs from obese mice appear depleted of miR‐141‐3p compared to EVs from wild‐type mice; miR‐141‐3p is a key in promoting insulin sensitivity in hepatocytes [39]. Treating AT with the EV biogenesis and release inhibitor GW4869 reduced the accumulation of miR‐141‐3p in hepatocytes (demonstrated via a co‐culture model), evidencing the pivotal role of EVs in facilitating the transport of miRNAs in cellular crosstalk.

**FIGURE 2:**
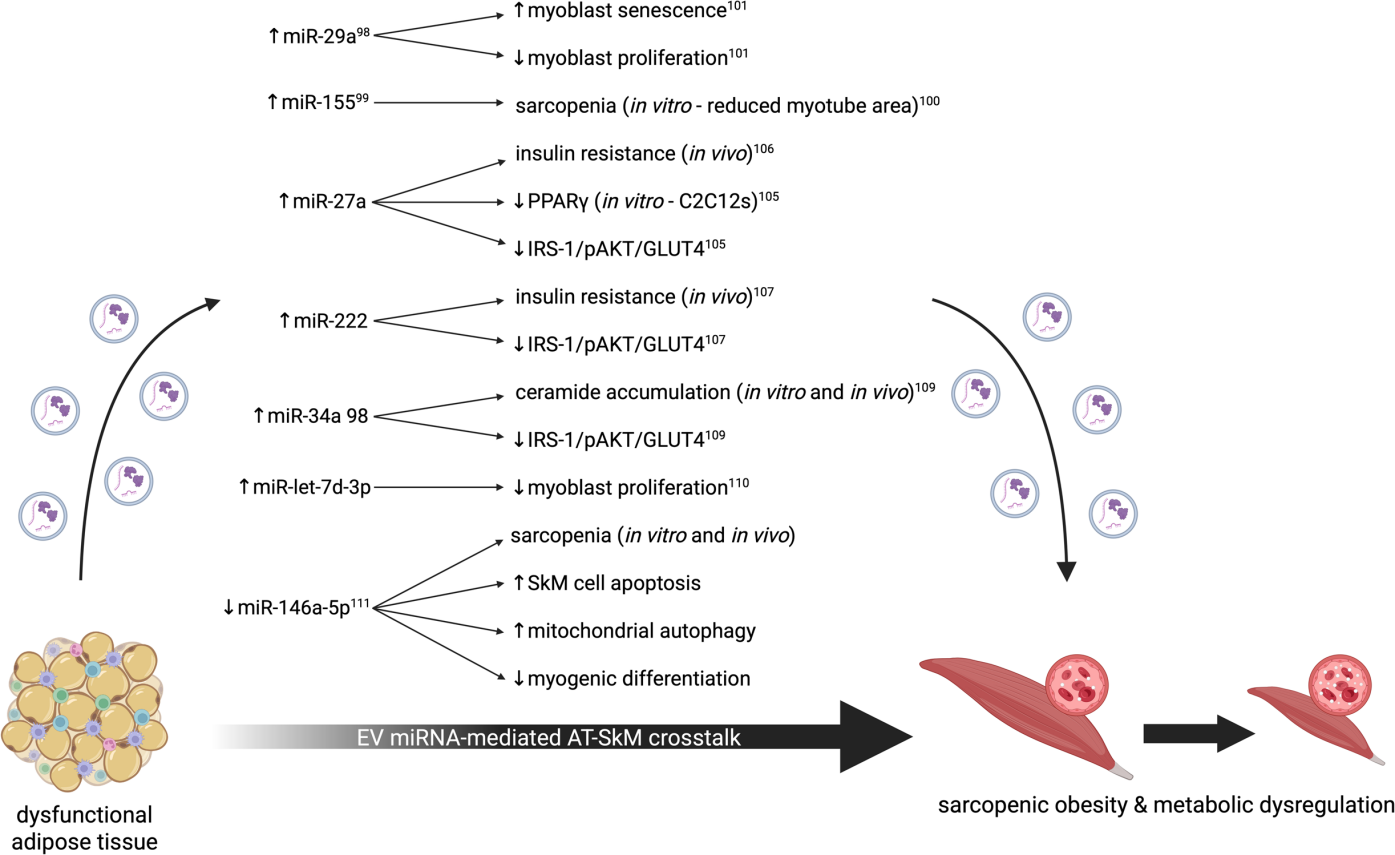
EV miRNA‐mediated AT–SkM crosstalk. EVs released from dysfunctional AT (due to age, obesity, and inflammation) contain miRNA cargo that appears to participate in crosstalk‐mediated sarcopenia and metabolic dysregulation in SkM. Created in BioRender. Macleod, M. (2025) https://BioRender.com/t09i755

Given that conditions associated with sarcopenic obesity are associated with altered EV miRNA cargo (see Section 3) and that EV‐associated transport of miRNA has the potential to mediate functional crosstalk both in vitro [39, 94] and in vivo [95, 96, 97], it is likely that EV‐associated miRNA cargo participates in AT–SkM crosstalk (Figure 2). Indeed, AT macrophage–derived EVs modulate insulin resistance in myocytes; with obesity, both miR‐29a and miR‐155 are upregulated in these EVs, suppressing PPAR*δ* and PPAR*γ* respectively and driving glucose intolerance through impaired GLUT4 expression [98, 99]. Other work has postulated a role for these miRNAs in SkM cellular senescence and atrophy, implying that miR‐29a and miR‐155 may participate in crosstalk to exacerbate metabolic dysregulation and sarcopenia with obesity [100, 101]. While miR‐29 has been shown to be mildly downregulated in the plasma of Chinese individuals with sarcopenia and frailty [102], the evidence is weak, driven by a small number of individual data points, and fails to account for significant differences in BMI and body fat percentage between the sarcopenic and non‐sarcopenic groups. However, miR‐29 is consistently upregulated in metabolic diseases in various tissues (including AT [103] and SkM [104]) and has been shown to induce insulin resistance both in vitro and in an in vivo mouse model [104]. miR‐27a has also been identified in negatively modulating PPAR*γ* and GLUT4 expression [105]. Palmitate treatment was used to induce hypertrophy in three T3‐L1‐derived adipocytes, increasing expression of miR‐27a in EVs from these cells. Myotubes differentiated from the murine myoblast cell line C2C12 were then treated with EVs from control or palmitate‐treated obese adipocytes; obese adipocyte–derived EVs enriched in miR‐27a inhibited PPAR*γ* and reduced GLUT4 and IRS‐1 expression, and AKT phosphorylation. However, in mice, exercise can prevent HFD‐induced elevations of EV‐miR‐27a and can alleviate HFD‐induced impairment of the IRS‐1/pAKT/GLUT4 pathway, both mechanisms by which insulin sensitivity is protected [106]. Other work confers a role for EV‐associated miR‐222 in driving insulin resistance in SkM tissue in vivo [107]. Mice fed a HFD show elevated miR‐222 in EVs in circulation and directly from gonadal AT, which the authors show downregulates the IRS‐1/pAKT pathway in muscle. Crucially, following surgical removal of gonadal AT, SkM insulin sensitivity was restored, implying an AT depot‐specific effect. Combining findings from Pan et al. [108] with work by Kukreti et al. [109] reveals a potential role for AT EV‐associated miR‐34a in modulating insulin signaling in SkM. miR‐34a is associated with adipocyte‐derived EVs, increases with obesity, and propagates AT inflammatory signaling by preventing M2 macrophage polarization [108]. miR‐34a expression is also increased in insulin‐resistant human myoblasts and aged murine SkM tissue and is associated with impaired IRS‐1/pAKT/GLUT4 signaling via inhibition of ceramide kinase and subsequent accumulation of ceramide [109]. Although no mechanistic work directly demonstrates miR‐34a functioning in AT–SkM crosstalk, this would be a plausible candidate for driving SkM insulin resistance in sarcopenic obesity given the previously discussed findings.

Although much research investigating EV miRNA‐mediated AT–SkM crosstalk addresses the effect on SkM insulin signaling, some miRNAs have been implicated in other SkM processes. For example, Sanz‐Ros et al. identified three key miRNAs (miR‐125b‐5p, miR‐let‐7c‐5p, and miR‐214‐3p) enriched in EVs from adipose‐derived stem cells that, when applied to senescent murine myoblasts, reduced senescence [95]. In vivo, these EVs restored a youthful phenotype (improved strength, motor coordination, and fatigue resistance) to aged mice at 14 and 30 days after administration, but this effect was transient and disappeared by 60 days. Furthermore, muscle fiber diameter and Type II fiber proportion (which generally decline with age) increased in EV‐treated animals versus control animals, showing potential for adipose‐derived EVs to induce structural age‐related changes in SkM. Additionally, Itokazu et al. showed that aged perimuscular AT EVs are enriched in miR‐let‐7d‐3p and that this reduces expression of the transcription factor Hmga2, leading to impaired proliferation of muscle progenitor cells [110]. The authors show that in vitro treatment of adipocytes with inflammatory cytokines (TNFα and IL‐1β) drives increases in EV miR‐let‐7d‐3p, implying that inflammageing modulates AT‐derived EV cargo involved in crosstalk with SkM. Recent work by Qin et al. presents detailed evidence of key EV miRNA‐mediated AT–SkM crosstalk [111]. The authors find an age‐associated decline in miR‐146a‐5p expression in both porcine and murine SkM tissue, which they correlate with increased atrophic markers. Mechanistic in vitro validation demonstrates a protective role for this miRNA in muscle, as treating C2C12 myotubes with a miR‐146a‐5p mimic reduces apoptosis, mitochondrial autophagy, and atrophic markers, while increasing markers of myogenic differentiation. These findings are reproduced in vivo via intramuscular injection of adipose‐derived EVs from control or miR‐146a‐5p knockout animals, with further mechanistic experiments implicating suppression of the E3 ligase Fbx32/MAFbx in rescuing the aged SkM phenotype.

Despite extensive and compelling work in this area, most research uses murine models. Critically, many miRNAs are not functionally conserved across mice and humans, and AT depots and composition differ significantly (reviewed previously [112, 113]). Given that the functional effects of EV‐associated miRNAs appear depot specific [35, 107], this potentially inhibits the translation of findings to humans and prevents the application of this work to developing therapeutic interventions to tackle sarcopenic obesity. Using human‐derived cells and tissues would enhance the translational potential of this field.

## 5 EV Protein‐Mediated AT–SkM Crosstalk

**FIGURE 3:**
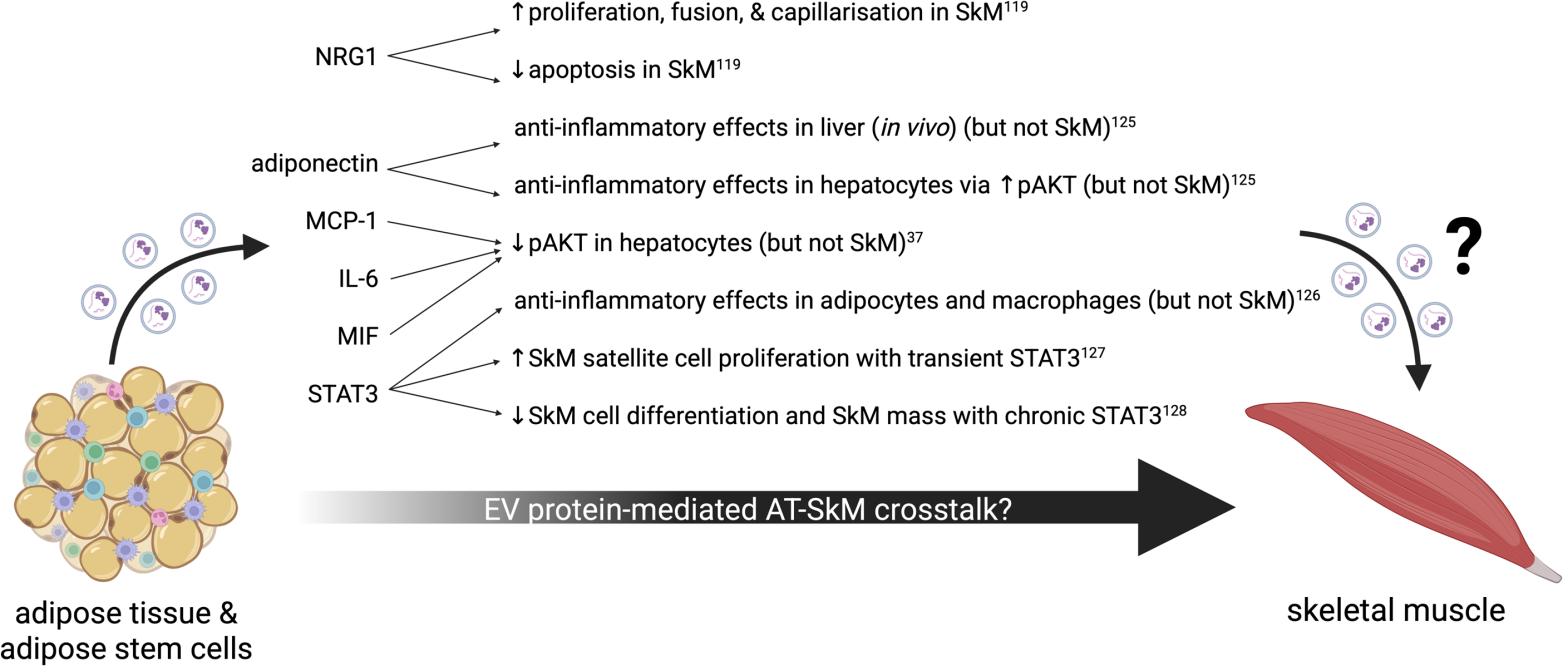
EV protein‐mediated intercellular crosstalk in sarcopenic obesity and metabolic dysregulation. EVs released from AT and AT‐derived stem cells contain protein cargo that has been, either directly or indirectly, linked to mediating effects in sarcopenic obesity and metabolic dysregulation by intercellular crosstalk. Created in BioRender. Macleod, M. (2025) https://BioRender.com/s73s341

EVs transport protein internally and bound to their surface [45], and it is widely accepted that EV protein cargo has the potential to modulate target cell physiology; for example, EV‐associated hexokinase 1 increases glycolytic activity of hepatocellular carcinoma cells [114], surface CD9 expression modulation functionally alters melanoma cell mitophagy [115], and P4HB in esophageal cancer–derived EVs promotes cachexia by inducing SkM cell apoptosis [116]. Mitchell et al. showed that the secretome of adipose‐derived mesenchymal stem cells presents differing protein profiles when comparing EV‐associated and soluble fractions and that these proteins independently modulate cellular processes to promote SkM regeneration [117]. Their work demonstrates that EV‐specific proteins (of which there are 301 that do not appear in the soluble fraction) have a unique and specialized role, rather than merely replicating the soluble, non–EV secretome fraction. Additionally, Hartwig et al. identified 897 proteins within primary human adipocyte‐derived EVs, emphasizing the sizeable contribution that EV‐associated proteins make to the total adipocyte secretome [118]. Despite this, work assessing the functional role of specific protein cargo in EV‐mediated AT–SkM crosstalk is scarce. To our knowledge, there is no published work describing a direct link between adipose‐derived EV protein cargo and functional changes in SkM in humans. However, work from our group shows that subcutaneous AT secretome (and the associated adipokines) from individuals with obesity impairs growth and nuclear fusion of primary human myotubes from older adults [30]. Given the reported synergistic yet independent effects of soluble and EV‐associated proteins from AT [117], it would be reasonable to hypothesize that EV protein cargo is also implicated in AT–SkM crosstalk (Figure 3).

The only paper to identify and functionally demonstrate the role of a specific EV‐associated protein in AT–SkM crosstalk used a murine model of hindlimb ischemia to demonstrate the regenerative potential of EVs from an adipose‐derived stem cell line [119]. In vitro and in vivo, adipose stem cell EVs induced protective effects on SkM (C2C12 myotube culture and C57BL/6J mice) by promoting proliferation, fusion, and capillarization, and preventing apoptosis. Subsequent proteomic analysis revealed neuregulin1 (NRG1) enrichment in these EVs; blocking NRG1 reduced the protective function of the EVs, likely because of modulation of the p38/MAPK pathway [120]. Pre‐treatment of EVs with trypsin (to cleave surface‐bound proteins) also impaired protective effects, implying that functional NRG1 is surface bound rather than internalized. However, no validation of EV integrity following trypsin treatment was performed. Additionally, EV protein cargo is altered in obesity [38]; therefore, analysis of whether EVs derived from obese adipose stem cells retain this reported protective effect on SkM would provide insight for muscle homeostasis and growth in sarcopenic obesity.

To date, no other work has directly demonstrated the role of EV‐associated proteins in AT–SkM crosstalk; however, some potential candidates mediate whole‐body metabolism and may modulate SkM. For example, Blandin et al. demonstrated that EV surface‐bound adiponectin modulates insulin sensitivity in hepatocytes (HepG2, a hepatoblastoma‐derived human cell line commonly used as an alternative model of human hepatocytes due to retention of the majority of normal cellular functioning of hepatocytes [121, 122, 123, 124]) via alterations to pAKT [125]. In vitro, EVs from lean visceral AT restore insulin sensitivity to palmitate‐treated hepatocytes, but EVs derived from obese visceral AT do not. Pre‐treatment of EVs derived from obese tissue with adiponectin restores their function to that of EVs derived from lean tissue, demonstrating a functional role of adiponectin via an EV‐mediated mechanism. In vivo administration of adiponectin‐enriched EVs prevented HFD‐induced gain of adiposity and reduced MCP‐1 expression in visceral AT and liver tissue, implying anti‐inflammatory effects. However, no effect was found in SkM. This work shows that EV‐associated proteins mechanistically modulate insulin sensitivity, but further work is required to validate this in SkM.

Kranendonk et al. found that the MCP‐1 content of subcutaneous AT EVs, and the IL‐6 and MIF content of visceral AT EVs, was negatively correlated with pAKT in hepatocytes [37]. Additionally, in hepatocytes, two distinct groups appear following EV and insulin treatment; pAKT is either increased or decreased, and the authors link this to EV adipokine content. However, this does not translate to the treatment of C2C12 myotubes, implying that EVs from the same individual impact the liver and SkM differently. A potential explanation for this is the low dose of AT EVs used in this work; hepatocytes and myocytes were treated with EVs derived from only 0.1 g of AT. Dosing treatments by EV number, rather than tissue weight, may elucidate more clearly the effects that these EVs have on the liver and SkM.

EVs derived from adipose stem cells have been shown to contain the protein STAT3, which can be functionally delivered to adipocytes and macrophages, promoting anti‐inflammatory phenotype shifts (browning of white adipocytes and M2 polarization of macrophages) [126]. This may be relevant for whole‐body metabolic health, but further work implicates STAT3 directly in SkM function. Although reports are mixed, transient activation of STAT3 may repair damaged muscle by promoting satellite cell proliferation, but chronically elevated STAT3, a downstream effector of IL‐6, prevents muscle cell differentiation and thus is detrimental to muscle function [127, 128]. Future work should seek to demonstrate whether adipose stem cell–derived EVs can functionally transport STAT3 to SkM, as this may represent a target for therapeutic modulation of SkM.

Further investigation of EV‐associated protein‐mediated AT–SkM crosstalk is required to elucidate the mechanisms of SkM atrophy and metabolic dysregulation and to identify candidate therapeutic targets for sarcopenic obesity. Comparative study of lean and obese AT EVs (visceral and subcutaneous depots) may reveal these targets.

## 6 EV Lipid Cargo

There is growing interest in EV lipid cargo profile and how lipidome composition may facilitate EV signaling. AT is a key in local and distal lipid metabolism [129], with ceramide accumulation (family of lipids) and diacylglycerols representing important mediators of insulin resistance [130]. However, ceramide and diacylglycerol uptake mechanisms remain elusive. The lipidome is complex with over 1000 lipid species; therefore, insulin resistance is likely modulated by multiple lipids [131]. For example, intramuscular long‐chain acyl‐coenzyme A is implicated in acute insulin resistance [132], with acylcarnitines also potentially contributing [133]. It has also been shown that omega‐3 fatty acids have the potential to partially ameliorate lipid‐induced insulin sensitivity independent of acylcarnitine accumulation [134].

It is feasible that one mechanism of lipid delivery and uptake is via EVs. Blandin et al. showed that the AT lipidome varies with obesity, and that although the EV lipidome varies similarly, there are additional changes in ceramide species and increases in phosphatidylinositol and sphingomyelin species in the adipose‐derived EVs, implying a selective sorting mechanism by which lipids are loaded into EVs in a BMI‐dependent manner [125, 135]. Altered adipose EV lipid cargo may be responsible for altered lipid accumulation in muscle, driving AKT inhibition and mitochondrial dysfunction [136]. Furthermore, mechanistic evidence from murine experiments shows that high palmitate diets, or palmitate treatment of cells, induce changes in mRNA expression of genes including MyoD1, MyoG, and IL‐6 [137]. EVs released from palmitate‐treated C2C12 myotubes were enriched with palmitate, implying that SkM can uptake palmitate, and package and release this via EVs. Crucially, C2C12 myotubes treated with palmitate, or with EVs released from palmitate‐treated C2C12 myotubes, demonstrated similar gene expression changes, evidencing an ability for SkM to both release and functionally uptake EV‐associated palmitate. Other studies have shown that EV surface‐bound lipids facilitate the biological function of non‐lipid EV cargo independent of direct signaling; for example, membrane lipids maintain EV structure and stability [138], potentially hold importance for the physiological integrity of EVs exposed to shear stress in circulation [139, 140], and facilitate binding and uptake at target cells [93].

In comparison to miRNA and protein cargo, little is published on the adipose EV lipidome, but a growing body of research is increasingly showing important EV lipidome functions, for example, linking EV‐associated ceramide with neurodegeneration [141]. The EV lipidome is thus an emerging field with a potential mechanistic role in AT–SkM crosstalk, holding promise as an area for future research.

## 7 Future Perspectives

Identification of AT‐specific EV surface markers would promote significant and rapid advancement in this field by facilitating the phenotyping and characterization of circulating EVs to determine the contribution of AT in physiologically relevant situations [135]. By doing so, the translational ability of in vitro and in vivo mechanistic work would be greatly improved. Research in this field commonly profiles circulating EVs (e.g., derived from plasma samples); thus, the ability to validate tissue origin would strengthen conclusions.

Additionally, an interesting yet relatively underexplored question is, aside from purported negative effects of obese adipose EVs, whether lean AT, or adipose‐derived stem cells (which decline with age and obesity [142]), release(s) EVs that exert protective or positive effects upon target tissues such as SkM. Some rodent studies, specifically those summarized in Section 5, imply that this may be the case [99], but further exploration may elucidate mechanisms of crosstalk regulation that inform the development of interventions in humans.

Aside from roles in glucose uptake and glycogen storage, SkM itself functions as an endocrine organ, releasing myokines and EVs in exercise‐ and obesity‐modulated states [84]. Although this review focuses on unidirectional EV crosstalk (from AT to SkM), future work could address the potential role of SkM‐derived EVs in modulating sarcopenic obesity and metabolic health via crosstalk with AT.

## 8 Conclusion

Crosstalk between AT and SkM holds a potentially crucial role in the pathophysiology of sarcopenic obesity and metabolic dysregulation. The previously discussed evidence, as depicted in Figure 2, implies a potential role for AT EV miRNA cargo in driving SkM insulin resistance, while also suggesting involvement in mediating sarcopenia. Furthermore, Figure 3 depicts current knowledge of protein‐mediated crosstalk between AT and SkM. To further elucidate the mechanisms of EV‐mediated AT–SkM crosstalk and to unveil directionality in the relationship between sarcopenic obesity and EV alterations, it will be necessary for greater standardization of EV methodology and increased utilization of in vivo animal models and in vitro human‐derived tissues.

The rapidly growing popularity of EV research is likely driven by their potential as targets and delivery tools in pharmaceutical interventions [143]. Given the debilitating effects of sarcopenic obesity, EV‐associated therapeutic targeting of dysregulated tissue crosstalk mechanisms holds promise for future treatment. Indeed, experimental work by Chen et al. and Zheng et al. has demonstrated the ability to modulate EV luminal cargo and surface proteins, altering functional consequences [144, 145]. These data are promising but still in its infancy. As detailed thoroughly by Klyachko et al. and Van Delen et al., some success has been achieved in loading EVs with therapeutic cargo and drug compounds, and thus clinical demonstration of the therapeutic use of EVs is currently in progress [146, 147]. An alternative promising approach appears to be direct pharmacological targeting of EV cargo or inhibition of EV uptake to modulate pathway activation in target tissues [148]. Given the role that EV‐mediated crosstalk may hold in sarcopenic obesity pathology, EV‐based therapeutics present a case for optimism. The rapid growth of the EV field and the concurrent increased push for rigor and standardization of EV research is promising. As this push continues, there are great advances to be made in the understanding of physiological tissue crosstalk in health and disease, hence the present review of current understanding in the contexts of sarcopenic obesity and metabolic dysregulation.

## Ethics Statement

This manuscript is a review of existing literature and thus does not contain primary clinical studies or patient data.

## Conflicts of Interest

M.M. is funded by the MRC AIM iCASE Doctoral Training Program with supplemental industrial support from AstraZeneca. E.T. and D.J.B. are employees of AstraZeneca. J.P., K.T., and S.W.J. declare no conflicts of interest.
